# A Methylcellulose Hydrogel as Support for 3D Plotting of Complex Shaped Calcium Phosphate Scaffolds

**DOI:** 10.3390/gels4030068

**Published:** 2018-08-11

**Authors:** Tilman Ahlfeld, Tino Köhler, Charis Czichy, Anja Lode, Michael Gelinsky

**Affiliations:** 1 Centre for Translational Bone, Joint and Soft Tissue Research, University Hospital Carl Gustav Carus and Faculty of Medicine, Technische Universität Dresden, 01307 Dresden, Germany; tilman.ahlfeld@tu-dresden.de (T.A.); Tino.Koehler@tu-dresden.de (T.K.); 2 Institute of Fluid Mechanics, Chair of Magnetofluiddynamics, Measuring and Automation Technology, Technische Universität Dresden, 01069 Dresden, Germany; charis.czichy@tu-dresden.de

**Keywords:** calcium phosphate cement, methylcellulose, 3D plotting, support, hydroxyapatite

## Abstract

3D plotting is an additive manufacturing technology enabling biofabrication, thus the integration of cells or biologically sensitive proteins or growth factors into the manufacturing process. However, most (bio-)inks developed for 3D plotting were not shown to be processed into clinically relevant geometries comprising critical overhangs and cavities, which would collapse without a sufficient support material. Herein, we have developed a support hydrogel ink based on methylcellulose (mc), which is able to act as support as long as the co-plotted main structure is not stable. Therefore, 6 *w*/*v* %, 8 *w*/*v* % and 10 *w*/*v* % mc were allowed to swell in water, resulting in viscous inks, which were characterized for their rheological and extrusion properties. The successful usage of 10 *w*/*v* % mc as support ink was proven by multichannel plotting of the support together with a plottable calcium phosphate cement (CPC) acting as main structure. CPC scaffolds displaying critical overhangs or a large central cavity could be plotted accurately with the newly developed mc support ink. The dissolution properties of mc allowed complete removal of the gel without residuals, once CPC setting was finished. Finally, we fabricated a scaphoid bone model by computed tomography data acquisition and co-extrusion of CPC and the mc support hydrogel.

## 1. Introduction

In the past, high-temperature extrusion based additive manufacturing, known as fused deposition modeling (FDM) was used to fabricate scaffolds with high diversity of shapes [1,2]. Low-temperature extrusion, called 3D plotting, was shown to combine the geometrical features of FDM with more advanced functionalities like inclusion of proteins and growth factors or bioplotting of cell-laden scaffolds with spatially defined cell distribution [3,4]. However, in contrast to FDM, until now, 3D plotting of either cell-laden bioinks or cell-free biomaterial inks rarely was shown for manufacturing complex shaped, volumetric constructs (>10 × 10 × 10 mm^3^) [5]. These shapes include critical overhangs which produce a bending moment which cannot be balanced by the main body of the scaffold and cavities which collapse due to gravity. Even the fabrication of constructs without flat side (created by deposition of material on the flat building platform of the printer) is impossible. Maybe, therefore, it is common until now that most publications show the fabrication of an ear-shaped model to demonstrate the capability of novel materials or printing technologies, but an ear displays neither a critical overhang nor a cavity, but has a flat bottom side.

First attempts to overcome shape limitations of 3D plotting were developed by usage of a support bath [6,7,8]. However, the support bath will interact with the main structure limiting the material variety of this method, as the interaction might cause needle clogging, hinder controlled post-processing, or even might bind physically to the main structure.

Multichannel plotting of a temporary supporting material together with the actual main ink could extend both, material variety and shape diversity of 3D plotted constructs. An ideal ink should comprise two functionalities: (i) the material ink acts as support for the main ink, preventing plotted strands from collapsing and (ii) the material ink acts as sacrificial material, which means that it is vanishing after post-processing and stabilization of the main scaffold. Additionally, material inks applicable for 3D plotting of support structures must not only meet the requirements mentioned, but also those of viscosity, plottability, and post-plotting stability. Within this manuscript, the term support ink is used, however the developed biomaterial ink also meets the requirement acting as sacrificial material.

Hydrogels have been developed for several applications of 3D plotting and their viscoelastic properties make them ideal for usage not just as bioink but also as cell-free support ink [9]. The ink mostly used for 3D plotting of support structures is Pluronic^®^ F127 (poloxamer 407—a synthetic, nonionic triblock copolymer). Previous studies investigated its use to create channel-like structures or sacrificial molds [10,11,12]. However, Pluronic^®^ F127 is also used to create bioinks acting as a main structure and is needed in high concentrations to show enhanced plotting properties [11,13,14]. Thus, there is a high demand on new, versatile applicable materials acting as support material.

Water-soluble methylcellulose (mc) could be a promising candidate. mc is a synthetic cellulose ether with methyl groups substituting hydrogen atoms of the cellulose molecules. It showed unique viscosity enhancement of aqueous solutions, as well as increased dissolution properties at decreasing temperatures (sol-gel transition was observed in a temperature range between 20–50 °C, depending on the molecular weight and addition of salts) [15,16,17]. Already in the 1940s, mc was investigated for medical use in ophthalmology [18] and furthermore is registered in the list of *Inactive Ingredients Search for Approved Drug Products* by the FDA [19], minimizing the risk of negative effects caused by possible residues at the main scaffold. Recently it was shown that pure mc is a promising material for extrusion-based printing [20].

For our study, we have chosen to use a ceramic calcium phosphate cement (CPC) as the main ink. CPCs are composites of inorganic precursors and an aqueous solution. In contact with water molecules, the setting process of CPC is initiated and, for example just like within this work, α-tricalcium phosphate is precipitating to nanocrystalline hydroxyapatite (HAp). Nonetheless, the usage of the classical cements does not allow fabrication by 3D plotting (or sometimes also referred to as robocasting), as the fast setting process leads to needle clogging. Therefore, organic carrier liquids were developed substituting the aqueous phase. They were shown to retard the hydroxyapatite precipitation reaction [21,22,23] and prevent needle clogging, enabling extrusion of CPC for a sufficient time to fabricate scaffolds. After extrusion to scaffolds and setting of the scaffolds either in water or water-saturated atmosphere, pure nanocrystalline and resorbable HAp scaffolds were achieved demonstrating high potential for bone applications in vivo [24,25,26].

Within this paper, we investigated the suitability of mc as support ink for CPC constructs, carried out by multichannel plotting of mc and CPC. We aimed to: (i) develop a mc paste fitting the support ink requirements; (ii) which was characterized for its extrusion and plotting properties. Based on these results, CPC constructs were fabricated with (iii) critical overhangs and (iv) cavities in order to enable fabrication of complex shaped CPC constructs, suitable as scaffolds for bone reconstruction or tissue engineering purposes.

## 2. Results

### 2.1. Development and Rheological Characterization of Methylcellulose Based Support Inks

Methylcellulose was dissolved in water in three different concentrations: 6 *w*/*v* %, 8 *w*/*v* % and 10 *w*/*v* %; therefore, the resulting support inks within this manuscript are referred to as mc6, mc8, and mc10, respectively. Higher concentrations of mc were excluded from this study, as it was not possible to achieve homogeneous gels with uniform viscosity. Furthermore, a low concentration was intended to be advantageous in terms of the sacrificial behaviour of mc inks. It was decided to dissolute mc at 4 °C to ensure full hydration of the mc chains. After complete dissolution, all three combinations, mc6, mc8, and mc10, were almost transparent, homogeneous and viscous pastes, which were used for further characterization.

**Figure 1:**
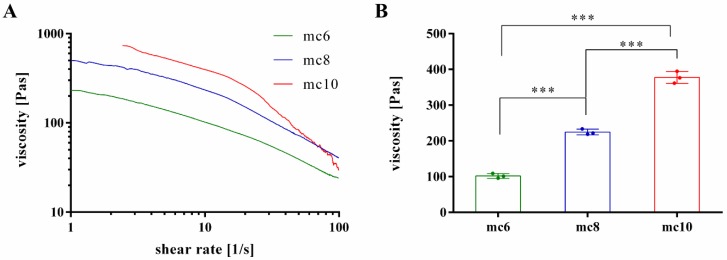
Rheological investigations on shear thinning behavior of mc6, mc8, and mc10 support bioinks. (**A**) Representative viscosity measurements at increasing shear rates; (**B**) Viscosity at a shear rate of 10 s^−1^ (mean ± SD, *** *p* < 0.001, *n* = 3).

To evaluate suitability of the pastes for 3D plotting, rheological properties at increasing shear rates were investigated (Figure 1A). All pastes showed shear thinning behaviour, which is the most important property of inks for extrusion-based application. As expected, the viscosity of mc pastes was highly dependent on the concentration; mc10 showed the highest viscosity, followed by mc8 and mc6. For further comparison of the mc pastes, the viscosity of the biomaterial inks was evaluated at a shear rate of 10 s^−1^, which is in our experience applicable to assess the plotting behavior of a material (Figure 1B) [27]. The viscosity of mc6 was 101.9 ± 6.6 Pa·s and significantly lower compared to mc8 (224.7 ± 7.8 Pas) and mc10 (377.5 ± 16.6 Pas).

**Figure 2:**
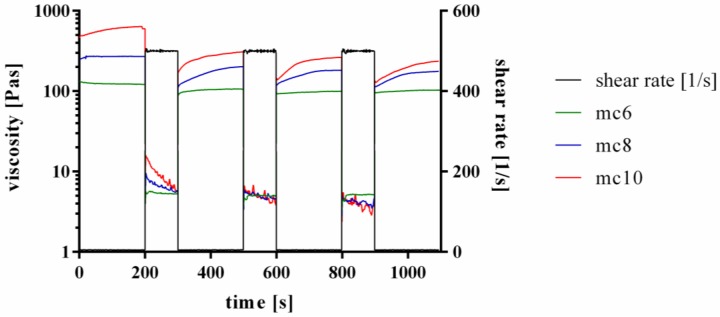
Representative curves of shear recovery experiments of mc6, mc8, and mc10 pastes.

Furthermore, the shear recovery of the support inks was tested, following the proposed protocols of Paxton and co-workers and Kesti and co-workers [13,28]. Therefore, firstly a low shear rate of 5 s^−1^ was applied for 200 s, followed by a hundred-fold higher shear rate of 500 s^−1^ for 100 s. This procedure was repeated two times. Representative curves are shown in Figure 2. All three tested inks, mc6, mc8, and mc10, respectively, showed a shear recovery behavior over time. The first low-shear phase displayed higher viscosities of the inks compared to the three following low-shear phases, indicating an initial and not reversible breakage of inner polymer structures after a high shear rate was applied to the pastes. However, the viscosity progress of the pastes at the second, third and fourth low shear region was similar, showing full recovery to the previous state without further significant molecular changes of the polymers after the first high shear rate was applied.

### 2.2. Extrusion Properties of Methylcellulose Support Biomaterial Inks

**Figure 3:**
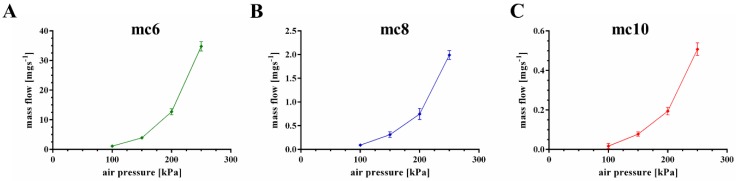
Mass flow of mc6, mc8, and mc10 pastes through a cylindrical needle (inner diameter 250 μm); consider the changes of the *y*-axes (mean ± SD, *n* = 3).

The extrusion properties were tested using a cylindrical needle with an inner diameter of 250 μm. Firstly, mass flow through the needle was evaluated as function of the applied air pressure (Figure 3). All three pastes showed increased mass flow with increased air pressure, but a significant (*p* < 0.001) higher mass flow of the mc6 paste was observed in comparison to mc8 and mc10. For example, at an air pressure of 200 kPa the mass flow of the mc6 paste was 12.72 ± 1.0 mg·s^−1^, whereas the mass flows of mc8 and mc10 were calculated as 0.75 ± 0.12 and 0.19 ± 0.02 mg·s^−1^. Based on these results, the following air pressure values were set for good extrusion with a plotting speed of 10 mm·s^−1^: mc6—100 kPa, mc8—200 kPa, and mc10—250 kPa.

**Figure 4:**
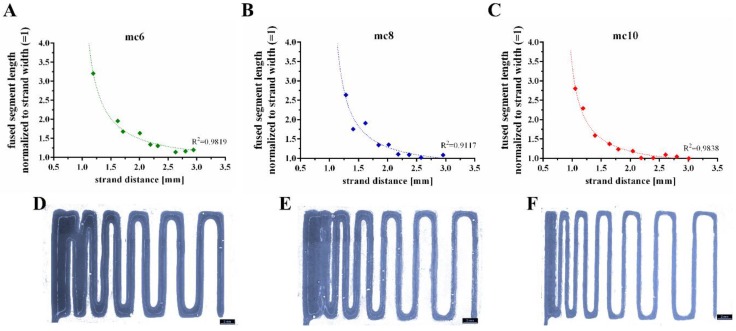
Representative fused filament tests of mc6 (**A**,**D**), mc8 (**B**,**E**), and mc10 (**C**,**F**) pastes.

Shape fidelity of the mc inks was evaluated by a filament fusion test, as proposed by Ribeiro and co-workers [29]. Shortly, three layers of the tested ink are plotted on top of each other. The layers are plotted as meanders and the strand distances increase continuously (Figure 4). By comparison of the fused segment length at the meanders to the strand width, the shape fidelity can be evaluated (the quotient should be near to 1.0). The resulting quotients of the filament fusion test are shown in Figure 4A–C as a function of the strand distance. For all pastes the shape fidelity was increased at higher strand distances. In comparison to the mc6 and mc8 biomaterial inks, mc10 displayed the smallest ratios at every strand distance, indicating higher shape fidelity. The measured strand widths of mc6, mc8, and mc10 were 1.20 ± 0.15 mm, 0.75 ± 0.05 mm, and 0.62 ± 0.04 mm, respectively; the differences between the strand widths are clearly visible in Figure 4D–F. This indicates collapses of the inks in *z*-direction with smallest collapse of the mc10 paste.

Based on the results of rheology and extrusion, mc10 was chosen to be the favorite support ink for 3D plotting of complex shaped CPC structures.

### 2.3. D Plotting of Calcium Phosphate Scaffolds with Overhanging Structures and Inner Cavities

**Figure 5:**
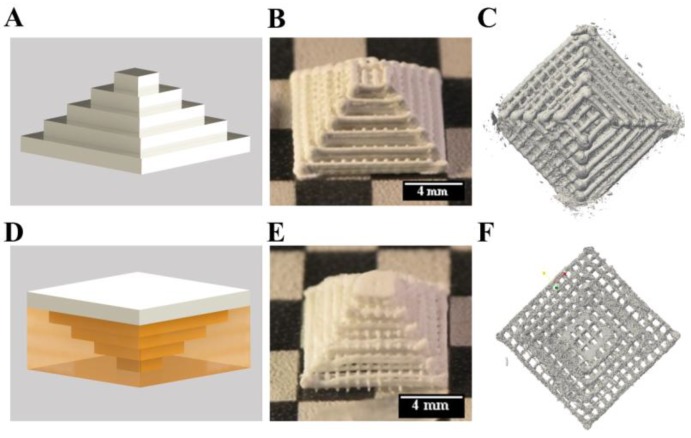
3D plotting of a Mayan pyramid with and without support structure. Principles (**A**,**D**) show the calcium phosphate cement (CPC) in white and mc in yellow. The upright and the inverted pyramids (**B**,**C** & **E**,**F**) revealed no changes of the inner structures, but at the borders.

Firstly, the shape fidelity of CPC constructs with overhangs was compared to structures of the same geometry, which were plotted without supporting material. The setup of the plotting process is shown in Figure 5A. A Mayan pyramid with macropores, consisting of CPC was plotted from the large bottom to the small top with a 610 μm needle. The layer structure was clearly visible (Figure 5B), macropores between the CPC strands were stable after setting and clearly visible in μCT (Figure 5C). The macroporosity calculated from micro computed tomography data was 69.6%.

Then, the pyramid was plotted in an inverted orientation displaying critical overhangs which would collapse without supporting material. The principle of plotting is shown in Figure 5D, by plotting the pyramid from the small top to the large bottom. The turned pyramid was plotted together with the mc10 ink (inner diameter of the needle: 610 μm) as support (Supplementary video SV 1). After plotting and post-processing, mc10 could be dried and removed mechanically without alteration of the CPC structure (Figure 5E). Micro-computed tomography revealed distinct macropores and the determined macroporosity was 66.8% (Figure 5F). Whilst the inner structure of the pyramids was comparable to each other without remarkable changes, the boundaries of the pyramids were different. The pyramid plotted without support structure in an upright orientation revealed roundish strands at the borders, while the pyramid plotted with support structure (upside down) had flatten borders and edges, probably as a result of the CPC pressing against the mc at their interface.

**Figure 6:**
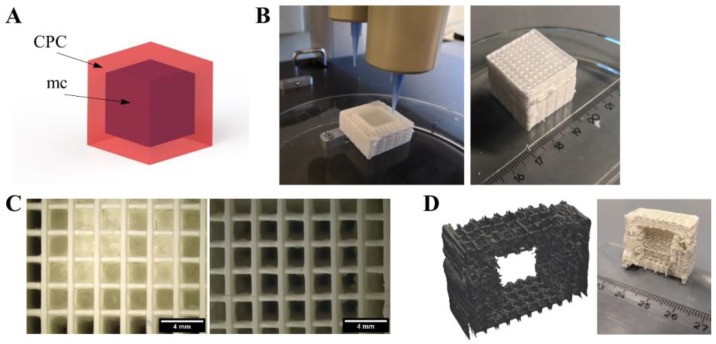
Generation of an inner cavity inside a CPC scaffold. (**A**) Principle of the CPC (red) structure with an inner cavity, filled with support material (mc, purple). (**B**) Fabrication and post-plotting appearance of the cubic structure with 410 μm needles. (**C**) After plotting (left), pores in *z*-direction of the CPC part are not interconnected but closed by mc at the interface of both materials. After methylcellulose vanished (right), the pores were clearly open and interconnected (scale bars 4 mm). (**D**) Virtual cut of micro computed tomography of the cube and photograph of the cut cube after mc vanished, revealing the cubical-shaped cavity inside the macroporous CPC structure. More images are provided in Supplementary Figure S1.

In contrast to a Mayan pyramid, there are structures which cannot be plotted without support. For example, big cavities within a construct would lead to a collapse of the layers plotted above. To demonstrate the suitability of the mc10 support ink for such applications, a cube-inside-cube structure was plotted as shown in Figure 6A. CPC (strand distance 2 mm) and mc10 were plotted successfully to a cuboid structure of 25 × 25 × 25 mm^3^. The inner cube plotted with mc10 (plotted without strand distance/macropores) had a volume of 15 × 15 × 15 mm^3^. After plotting, stereomicroscopical images showed clearly the presence of the non-porous mc10 cube (Figure 6C). Afterwards, the biphasic structure was incubated in humid atmosphere for 3 d, allowing the CPC to set to nanocrystalline HAp. Then, the biphasic scaffold was incubated in distilled water over night at 4 °C. Stereomicroscopy evidenced, that mc10 vanished (Figure 6C) and the pores in *z*-direction, being closed by mc before, were completely open. Micro-computed tomography and cutting of the CPC structure revealed that the inner cube, which now could be referred to as cavity, preserved the shape of the previous mc cube without collapse of the CPC layers above (Figure 6D). Additional images demonstrating the achieved even surface of CPC are shown in Supplementary Figure S1.

### 2.4. D plotting of a Clinically Relevant Structure

**Figure 7:**
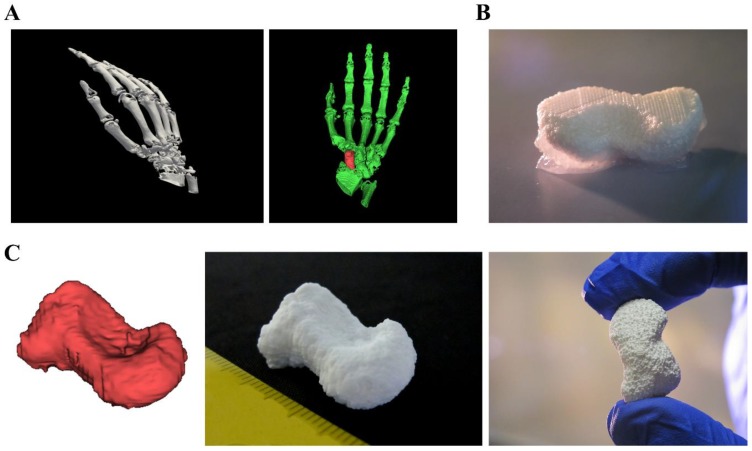
Fabrication of a scaphoid bone from clinical CT data. (**A**) 3D rendered human right hand and selection of the scaphoid by usage of 3D rendering software. (**B**) 3D multichannel plotting of the scaphoid bone consisting of CPC with mc10 as support ink. (**C**) Comparison of the stl-file and the real plotted structure with the characteristic shape of the human scaphoid.

Plotted CPC structures show great potential for scaffolds in regenerative bone applications. Until now, CPC plotting was already further developed concerning utilization of different material combinations, growth factor loading or integration of live cells into the fabrication process [30,31,32]. Utilization of mc10 as support material opens the window to plot clinically relevant shapes, fitting to patient-specific anatomical structures or individual defects. To show the potential of CPC for these purposes, fabrication of a human scaphoid bone was chosen as example (Figure 7). For this, open source software (Invesalius 3.1 [33]) was used to reconstruct and separate the scaphoid bone from a set of CT data (Figure 7A). The resulting stl-file was transferred to the BioScaffolder 3.1 software generating the support structure. Afterwards, CPC and mc10 were plotted together layer by layer (Figure 7B shows CPC in white and mc10 appearing opaque) by usage of needles with an inner diameter of 230 and 250 μm, respectively. Finally, the plotted construct demonstrated high shape equivalence to the stl-file and the characteristic curvature of the anatomical correct scaphoid (Figure 7C).

## 3. Discussion

Methylcellulose was investigated for its use as biomaterial ink for 3D plotting. A concentration of 10% was convenient to act as support material for plotted structures consisting of a calcium phosphate cement.

Generally, there are two main strategies to apply support to 3D plotted structures: utilization of a supportive bath, which later on gets removed or, as used within this study, the co-extrusion with a temporary support material. For example, the usage of a nanoclay support bath successfully was shown by Jin and co-workers [7]. However, the hydrophilic character of the bath is not applicable for calcium phosphate cements, as the setting reaction would start immediately in the plotting needle, which would result in fast needle clogging. Therefore, an alternative was shown by Miranda and co-workers, who successfully extruded a calcium phosphate cement into a hydrophobic oil-bath, preventing the fast setting reaction and needle clogging [34]. However, the oil bath properties would impair the possibilities of integration of biological sensitive factors and inhibit the inclusion of cells into the plotting process. Thus, co-extrusion of the support is most promising for 3D plotting of complex shaped, anatomical relevant CPC structures. Although the mc10 ink was especially developed for the purpose of support ink for CPC scaffolds, it should be applicable for several other cell-free and cell-laden inks, which act as main structure.

In this study, extrusion of mc6, mc8, and mc10 support inks was performed at room temperature, without heating or cooling the plotting needle. Due to its thermo-reversible properties, plotting and post-processing temperatures influence the behaviour of mc crucially [20,35]. Representative temperature sweep experiments of all three materials are shown in Supplementary Figure S2. For all inks, the sol-gel transition was above 37 °C. Thus, mc was in non-crosslinked state during both, extrusion and post-processing.

The plotting properties of the tested mc support inks were strongly dependent on their concentration. Especially mc10 and mc8 showed good printability and furthermore shape fidelity after extrusion, whereas plotted mc6 strands tended to collapse. The enhancement of shape fidelity of materials for 3D plotting was shown before in blends with alginate or a nanoclay-alginate composite [27,36]. Pure cellulose, or derivatives like bacterial nanocellulose were used to enhance viscosity and shape fidelity of 3D plotted scaffolds and cell-laden constructs [37,38,39]. However, the solubility of mc makes it an optimal material as support ink, which can vanish at a defined time point.

Herein, we have proven the suitability of co-plotting mc as support material for CPC constructs. Rheological and extrusion properties were investigated, showing high applicability for extrusion-based additive manufacturing. The viscosity of mc was increasing with increasing concentration. Further development of mc-based support inks could involve the presence of additives in the support paste. The gel point, and thus the gel strength/viscosity of mc can be adjusted by additives like sucrose, propylene glycol, or glycerin, which should impact the dissolution properties [40]. Recently, it was shown, that cationic dodecyltrimethylammonium salts (bromide, DTAB) was lowering the gel strength of mc [41]; also the addition of salts to mc was shown to change the transition temperature, and thus the gel point [17,35,42,43]. Cochis and co-workers investigated the effect of the extrusion temperature of salt-doped mc for extrusion-based applications [20]. This principle could help to further control the removal of plotted mc support structures.

There are two mechanisms for removal of mc10 from the main structure: Mechanical action or dissolving of the support ink. In case of the pyramids, mc10 was removed mechanically without damaging the CPC main structure. However, this method could harm the plotted main structure if it is mechanically unstable (like filigree structures or hydrogels) and cannot be applied to more complex geometries like cavities. Moreover, residues of mc are likely to remain. Therefore, the hydration process of mc should be the favorable method to remove mc after the main structure is post-processed and stable.

Herein, we successfully have developed a support ink based on methylcellulose, specifically applicable for a self-setting CPC. mc10 has comparable thermoresponsive properties like the commonly used Pluronic^®^ F127 (so it can be easily dissolved at 4 °C within a few hours), but additionally can dissolve at 37 °C, as it is still a non-crosslinked sol. For example, mc was shown to be released out of an alginate matrix at 37 °C, without harming cell viability of embedded cells [36]. In a recent study of our group, we have observed, that mc dissolution out of an alginate matrix at 37 °C is ongoing over 21 days and release kinetics can be influenced by control of the molecular weight [44] or, as shown for pure mc, by addition of several salts [17]. This is advantageous for several future applications, either for plotting-then-seeding or bioplotting of cells: mc-based support inks can dissolve without affecting cell viability at cell culture conditions (37 °C). Moreover, the utilization of mc as new support material opens up new fabrication methods; for example, some inks are based on Pluronic^®^ F127 [14], and might need another material as sacrificial support structure which can be dissolved at temperatures at which Pluronic^®^ F127 is stable.

For the first time, CPC was plotted with a support ink to obtain complex shaped constructs of clinical relevance. In the past, complex shaped calcium phosphate scaffolds were manufactured by powder printing [45,46,47], or selective laser sintering [48] with the powder bed automatically also working as stabilizing support. Calcium phosphate scaffolds, fabricated by stereolithography, did not collapse, due to the stabilizing effect of the surrounding resin, before the resulting scaffolds were sintered [49,50]. Furthermore, calcium phosphate scaffolds were additively manufactured by indirect printing [51], lithography-based ceramic manufacturing [52] or robocasting [34]. All of these methods have in common that combination with biologically sensitive proteins is difficult and the inclusion of cells into the fabrication process is impossible. In contrast, extrusion of CPC is conducted in mild conditions (at room or physiological temperature, without any organic solvents involved or energy-intensive post-processing steps etc.). This enables fabrication of CPC together with temperature sensitive substances even living cells [26,31,32,53]. Therefore, 3D plotting of CPC with support material does not only allow creation of complex shaped structures or implants, but also enables biofunctionalization of calcium phosphate implants in a spatially defined manner, which is superior to most other additive manufacturing methods.

Firstly, the influence of plotting a support structure together with a CPC main structure was evaluated. The inner structure of the tested pyramids was not affected, whereas the outer borders were changed distinctly. The pyramid plotted without support comprised more roundish strands, as it is expected for strands at a surface of every plotted scaffold. In contrast, the pyramid plotted with mc10 as support ink in reversed orientation revealed flattened strands at the interface to mc. Both, roundish and flattened strands are approximations of the real geometry of the pyramid. In the case of a possible implant, there are neither right angles nor complete convex surfaces. Therefore, the geometry of a single strand might never mimic the real geometry of a human bone specimen. As the resorption of the nanocrystalline HAp by osteoclasts is part of the natural bone remodeling process [54,55], the surface of plotted CPC implants will change over the whole healing time; thus, the strand surface does not necessarily have to reproduce the real surface of the bone completely.

However, the rough geometry of anatomical structures can be plotted: the example of the human scaphoid bone showed sufficient shape reproduction in comparison with the three-dimensional image file. Parameters influencing the plotted shape were on the one hand the resolution of the computed tomography data set and the precision of the generation of the stl-file. On the other hand, the strand distance and orientation, the used needle diameters for extrusion of CPC and mc and plotting properties like the layer thickness, applied air pressure and plotting velocity influence the outcome significantly. For CPC, these parameters were optimized in our group, achieving filigree strands and scaffolds with high inner complexity [53].

Crucially, CPC scaffolds could be fabricated containing large inner cavities. Such closed inner pores can only by generated by additive manufacturing methods. The dissolution properties of mc allowed fast vanishing of the internal mc cube after CPC was set (3 days), leaving behind a cavity of the desired cubic shape. This might enable the fabrication of scaffolds with highly controlled (macro)porosity to improve the ingrowth of cells and vascular structures, both accepted as main challenges in bone tissue engineering.

## 4. Conclusions

The usage of a newly developed methylcellulose-based support ink was sufficient to enable fabrication of structures with critical overhangs and to produce controlled cavities inside a CPC construct. For the first time, it was shown that CPC can be used to fabricate constructs of any clinical and anatomical relevant shape by 3D plotting. Furthermore, a human scaphoid bone model of high shape-fidelity could be produced. Further work should concentrate on more control of the dissolution properties of mc, which basically could be tailored for any application by addition of gel-strengthening and gel-weakening substances. The principle of biphasic plotting of the support ink and CPC will facilitate the production of patient-individual HAp implants, fabricated by 3D plotting.

## 5. Materials and Methods

### 5.1. Preparation of Plotting Paste

CPC was manufactured by InnoTERE GmbH (Radebeul, Germany). For the support material, methylcellulose powder (M0512, Sigma, St. Louis, MO, USA, molecular weight ≈ 88 kDa, 4000 cP) was dissolved for 12 h in deionized water at 4 °C in different concentrations (6 *w*/*v*%, 8 *w*/*v*% and 10 *w*/*v*%, respectively). To ensure full binding of water to mc, solutions were stirred every 2 h. The resulting support inks are referred to as mc6, mc8, and mc10.

### 5.2. Rheology

Rheological examinations of the mc pastes were performed with a plate-plate rheometer (Rheotest RN 4, Medingen, Germany; *d* = 36 mm, distance of the plates 0.1 mm) at 25 °C. Shear thinning was characterized by increasing the shear rate from 0–100 s^−1^ over 1200 s (increment 0.08 s^−1^) and viscosity was measured (*n* = 3). Shear recovery of the mc pastes (*n* = 3) was characterized orientating at the protocols proposed by Paxton and co-workers and Kesti and co-workers [13,28]. Firstly, a constant shear rate of 5 s^−1^ was applied for 200 s, followed by a hundred-fold higher shear rate of 500 s^−1^ for 100 s. This procedure was repeated two times. Then, again a shear rate of 5 s^−1^ was applied for 200 s. For the whole time of observation, viscosity was measured and shear recovery was obtained. Stress ramps of mc6, mc8, and mc10 were performed to evidence flowing of the inks at high shear rates (data not shown).

### 5.3. Mass Flow and Filament Fusion Test

The mass flow of mc6, mc8, and mc10 was conducted through a conical needle with an inner diameter of 250 μm (Globaco, Rödermark, Germany); needle and plate were at room temperature. The inks (*n* = 3) were allowed to flow through the needle for 50 s at different air pressures (100, 150, 200, 250 kPa), then the mass of the extruded volume was measured. Resulting mass flow $\dot{m}$ was calculated:

$$\dot{m}=\frac{m}{50\;s}$$

Filament fusion tests of the mc inks (*n* = 3) were performed following the protocol of Ribeiro and co-workers [29]. Shortly, three layers of the inks were plotted on top of each other. Each layer consisted of meandering strands with increasing strand distances. Immediately after plotting, the scaffolds were imaged by a stereo light microscope (Leica M205 C equipped with DFC295 camera, Wetzlar, Germany). Resulting images were analyzed by Fiji Image software [56] and actual strand distance, fused segment length and strand width were obtained from the data. The quotient of segment length to strand width was evaluated as function of the strand distance.

### 5.4. Fabrication of Volumetric Constructs and Post-Fabrication Treatment

Both, CPC and mc support inks, were filled into cartridges and plotted at room temperature (needle and plate) to scaffolds (sizes given in Figure captions) utilizing a three-axis multichannel plotter (BioScaffolder 3.1, GeSiM mbH, Radeberg, Germany). The needle (Globaco) dependent plotting parameters are summarized in Table 1. After plotting, CPC was allowed to set in water-saturated atmosphere (>95%) for 3 days. Afterwards, residual mc of biphasic CPC-mc constructs was dissolved in water at 4 °C.

### 5.5. Microcomputed Tomography

Micro X-ray computed tomography was conducted by a custom-built setup called TomoTU of TU Dresden, Institute of Fluid Mechanics, Chair of Magnetofluiddynamics, Measurement and Automation Technology. The grey value images of the sample were generated with 0.5° angular increment (720 images in total) for a tube current of 170 μA and an acceleration voltage of 90 kV. The size of the photodiode array and therefore of the images was 2304 × 2940 (vertical x horizontal) pixel. A magnification of 3.55 was chosen, which resulted in a resolution of 1 pixel = 13.95 μm. Reconstructions of the 3D-images was created by grey value analysis (threshold = 120) with Paraview 5.5 software [57]. Macroporosity of the scaffolds was determined by grey value analysis of the reconstructed image stacks (threshold = 120).

## Figures and Tables

**Table 1:** Plotting parameters of CPC and mc support inks.

Material Ink	Needle Size [μm]	Air Pressure [kPa]	Printing Speed [mm·s^−1^]	Layer Thickness [μm]
CPC	230	250	10	120
	410	200	10	260
	610	150	10	360
mc6	250	100	10	120
mc8	250	200	10	120
mc10	250	250	10	120
	410	180	10	260
	610	130	10	360

## Supplementary Materials

Supplementary materials can be found at http://www.mdpi.com/2310-2861/4/3/68/s1.
