# Supplementary material for: A Methylcellulose Hydrogel as Support for 3D Plotting of Complex Shaped Calcium Phosphate Scaffolds
Source: Gels. 2018 Aug 11;4(3):68. doi: 10.3390/gels4030068 (PMC6209251; doi:10.3390/gels4030068)
Supplement: Supplementary file 1 [file gels-04-00068-s001.zip › Supplemental.docx]

Supporting information

A methylcellulose hydrogel as support for 3D plotting of complex shaped calcium phosphate scaffolds

Tilman Ahlfeld^1^, Tino Köhler^1^, Charis Czichy^2^, Anja Lode^1^* and Michael Gelinsky^1*^

1. Centre for Translational Bone, Joint and Soft Tissue Research, University Hospital Carl Gustav Carus and Faculty of Medicine, Technische Universität Dresden, Dresden, Germany
2. Institute of Fluid Mechanics, Chair of Magnetofluiddynamics, Measuring and Automation Technology, Technische Universität Dresden, Dresden, Germany

- Correspondence: michael.gelinsky@tu-dresden.de; Tel.: +49-351-4586694, anja.lode@tu-dresden.de; Tel.: +49-351-45816692


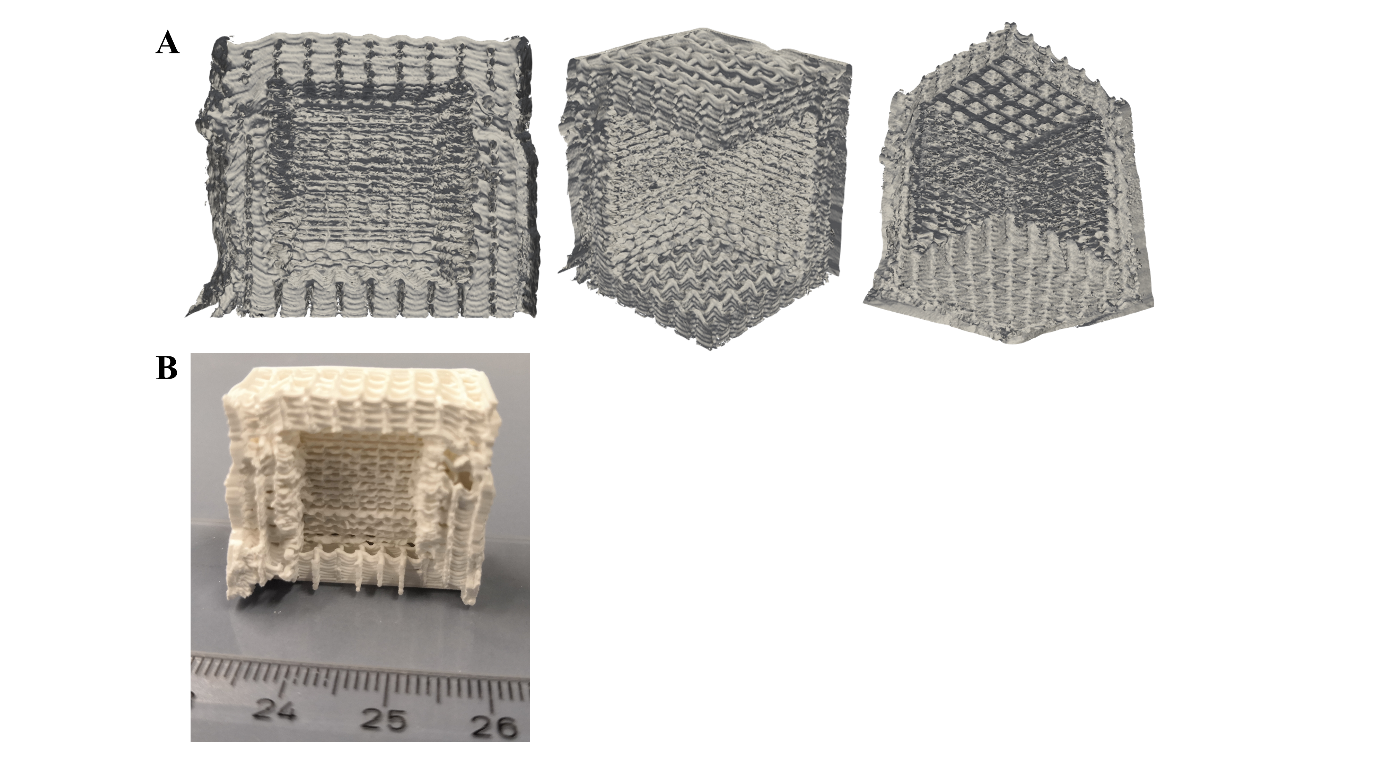


Supplemental figure S1: (A) Virtual cuts of reconstructed cube-inside-cube structure, clearly showing the shape of the inner cube was maintained after mc was released. (B) Image of the real cut scaffold.


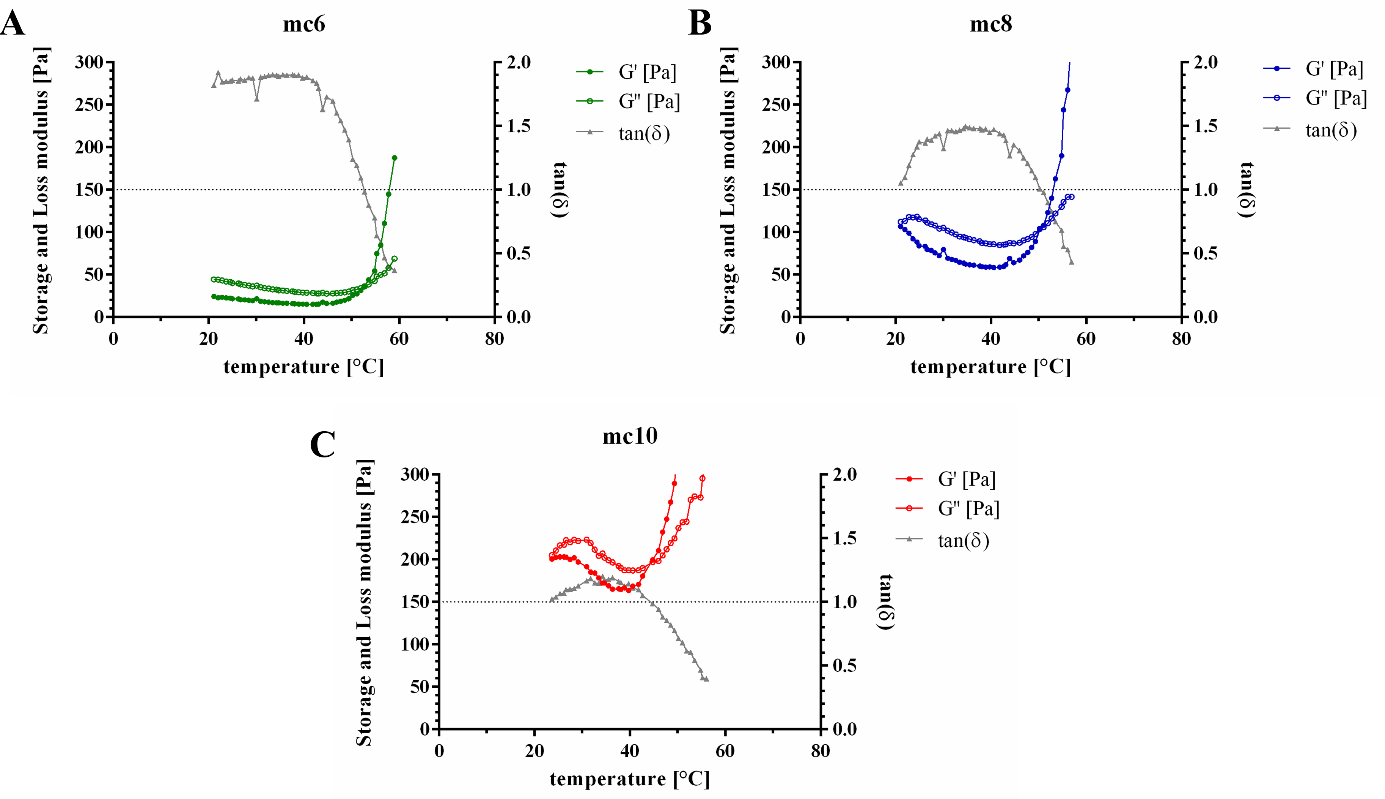


Supplemental figure S2: Representative curves of oscillatory temperature sweep measurements of (A) mc6, (B) mc8 and (C) mc10 support inks. Sol-gel transition was observed at temperatures of 51.0-52.6 °C for mc6 and mc8 and 43.1-43.9 °C for mc10.
